# Generous Leaders and Selfish Underdogs: Pro-Sociality in Despotic Macaques

**DOI:** 10.1371/journal.pone.0009734

**Published:** 2010-03-17

**Authors:** Jorg J. M. Massen, Lisette M. van den Berg, Berry M. Spruijt, Elisabeth H. M. Sterck

**Affiliations:** 1 Behavioural Biology, Utrecht University, Utrecht, The Netherlands; 2 Ethology Research, Biomedical Primate Research Centre, Rijswijk, The Netherlands; Georgia State University, United States of America

## Abstract

Actively granting food to a companion is called pro-social behavior and is considered to be part of altruism. Recent findings show that some non-human primates behave pro-socially. However, pro-social behavior is not expected in despotic species, since the steep dominance hierarchy will hamper pro-sociality. We show that some despotic long-tailed macaques do grant others access to food. Moreover, their dominance hierarchy determines pro-social behavior in an unexpected way: high-ranking individuals grant, while low-ranking individuals withhold their partner access to food. Surprisingly, pro-social behavior is not used by subordinates to obtain benefits from dominants, but by dominants to emphasize their dominance position. Hence, Machiavellian macaques rule not through “fear above love”, but through “be feared when needed and loved when possible”.

## Introduction

Altruism remains one of the major mysteries in evolution. Although reciprocal altruism [1] has been found in animals [2], genuine altruism, defined as a costly act that confers benefits on non-kin regardless of reward prospects, is considered uniquely human [3]. However, pro-social preferences to deliver food to unrelated individuals at no or very low cost were also reported for the common marmoset [4], a primate species that, similar to humans, shows a cooperative breeding system [5]. Consequently, it was suggested that pro-sociality may result from convergent evolution among cooperative breeders [4]. This hypothesis may not be tenable, since a recent study failed to show pro-social behaviour in another cooperatively breeding primate, the cottontop tamarin [6]. Moreover, subsequent studies also showed pro-sociality in non-cooperative breeding primates such as capuchin monkeys [7], [8] and bonobos [9]. This led to the alternative suggestion that pro-sociality is an ancestral trait among primates [8]. However, results of chimpanzees are inconsistent, and depending on the tests used chimpanzees do not [10]–[12] or do [13] show pro-social behaviour. These inconsistencies suggest that pro-social behavior may not be fully expressed among more despotic primate species. In line with these suggestions, it has been argued that human egalitarianism coevolved with pro-sociality [14]. Therefore, it is expected that despotism hampers pro-social behavior. However, this hypothesis remains to be tested experimentally.

Here we intend to test this proposition. We examined whether long-tailed macaques (*Macaca fascicularis*) behaved pro-socially towards conspecifics, without incurring costs to self. Long-tailed macaques belong to the family of Cercopithecidae. In contrast to most other primate species, it is considered easy to detect a clear dominance hierarchy in this family of primates [15]. Furthermore, long-tailed macaques are primates that have low social tolerance and a large dominance asymmetry. Therefore, within the genus Macaca they are considered a despotic species with a steep linear hierarchy [16]. Moreover, among these macaques kin relationships are important especially for females, and related individuals obtain neighboring, but clear, dominance ranks through mutual support [17]. Therefore, in our experiment we distinguish kin versus non-kin. Furthermore, among these macaques there are two opposite ways in which dominance rank may affect pro-social behavior: 1) Subordinates may act pro-social to those higher in rank, similar to grooming up the dominance hierarchy [18], [19], with possibly either tolerance or future support as a result. 2) High-ranked individuals' generosity may be a strategy to either enhance or maintain their status [20], [21].

## Results

**Figure 1:**
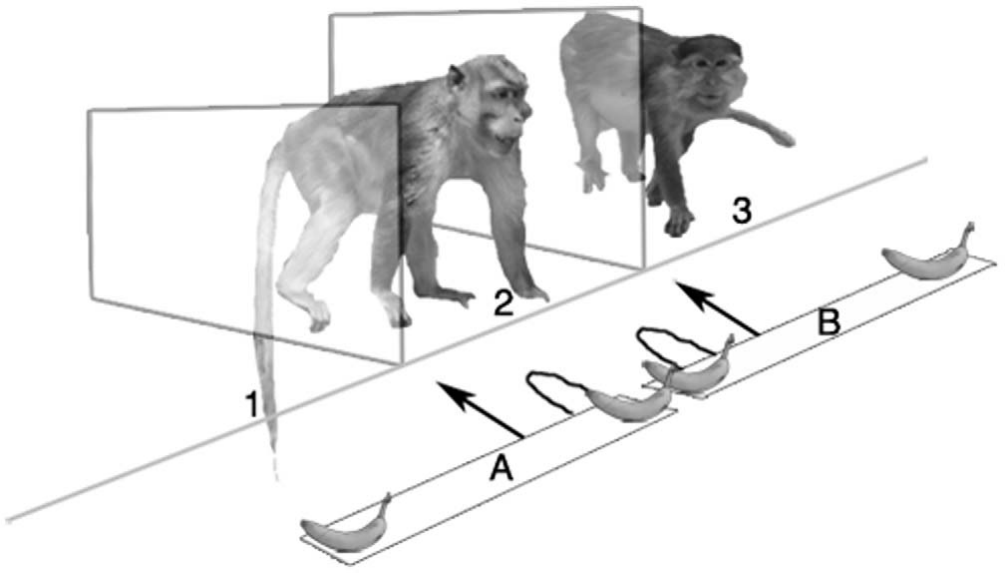
Drawing of two monkeys in the test setting. The drawing shows the subject in the middle compartment having the choice between either granting itself and its partner (in compartment three) access to a banana (choice B, the ‘pro-social’ choice), or granting only itself access to a banana and leaving a banana in front of an empty compartment (compartment one) (choice A, the ‘a-social’ choice).

In this study twenty captive long-tailed macaques from the same social group participated. They were placed alone in a test compartment, located between an empty test compartment and a test compartment occupied by another macaque (Figure 1). To avoid bargaining for sexual services [22], partners were always of the same sex. Test-setting and apparatus were almost identical to those used in one chimpanzee study [11]. The subject macaque was given the choice between two slides, each baited with two slices of banana. By pulling on one slide, the subject would gain access to one slice of banana, while the second slice was out of reach in front of the empty test compartment (choice A). Pulling on the second slide also allowed the subject access to only one slice of banana, and in addition the second slice came within reach of the individual in the adjacent cage (choice B). As both choices involved the same cost and benefit for the subject, but the second choice also involved a benefit for adjacent individual, we define the second choice as pro-social and the first as a-social. The preference for the ‘pro-social’ choice was compared to the control condition in which a side preference was measured when both adjacent cages were empty. In the test condition, partners were intentionally placed on the opposite side of the side preference measured in the control condition and consequently, subjects had to deviate from their initial side preference to be pro-social. Ten subjects, nine females and one male, were tested twice: both with a kin and a non-kin partner in the adjacent test compartment. Ten additional subjects, lacking same sexed kin, were tested with non-kin partners only.

**Figure 2:**
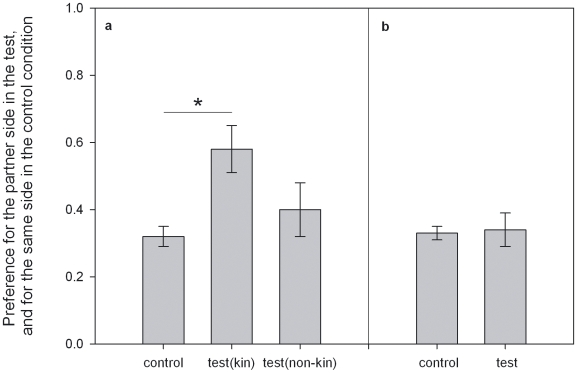
Pro-social preferences: **a. Pro-sociality and kin.** Mean preference ± s.e.m. for the partner-side of all individuals tested with their kin (n = 10) in the test condition, the mean preference ± s.e.m. for the same side of the same individuals in the control condition and the mean preference ± s.e.m. for the partner-side of the same individuals when tested with non-kin. **b. Pro-sociality and non-kin.** Mean preference ± s.e.m. for the partner-side of all individuals tested with non-kin (n = 20) in the test condition and the mean preference ± s.e.m. for the same side of the same individuals in the control condition * indicates a difference at the p<0.05 level (exact Wilcoxon signed ranks test).

First, our results show that five long-tailed macaques act significantly pro-socially towards kin (Chi square tests on each individual's choices: p<0.05), and one individual tends to do so (p<0.01). Moreover, overall their preference for the partner side when tested with a kin partner was significantly higher then their preference for the same side in the control condition (Kin: Test vs. Control; Wilcoxon signed ranks test: T^+^ = 4, n = 10, p_exact_ = 0.014)(Figure 2a). Since nine our of ten pairs concerned females, this in particular indicates that females are pro-social towards their female kin. Secondly, four out of the twenty individuals tested with a non-kin partner also acted significantly pro-social, while three were significantly the reverse of pro-social, or a-social (i.e., they differed from original side preference such that they withheld their partners access to food) (Chi square tests on each individual's choices: p<0.05), and two individuals tended to act a-social (p<0.01). Nonetheless, no overall significant difference between the preference for the partner side in the test and the same side in the control condition was found among non-kin (Wilcoxon signed ranks test: T^+^ = 98, n = 20, p_exact_ = 0.914)(Figure 2b). Consequently, the pro-social tendency towards kin partners was significantly higher then the pro-social tendency towards non-kin partners (Wilcoxon signed ranks test: T^+^ = 41, n = 10, p_exact_ = 0.031)(Figure 2a).

**Figure 3:**
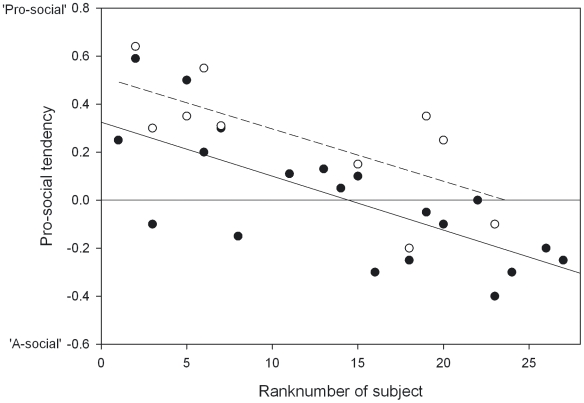
Pro-social tendency and rank. Pro-social tendency (difference between the preference for partner side in the test condition and the preference for the same side in the control condition) and absolute rank number (nr 1 is the alpha male) of all subjects towards kin (open circles and dotted line) and non-kin (closed circles and full line). Lines indicate linear regressions significant at the p<0.05 level.

Among non-kin, high-ranking individuals (with a low rank number) grant their partner access to food, whereas low-ranking individuals deny their partners access to food, which is demonstrated by a significant linear regression of pro-social tendency with dominance rank (t = −4.689, β = −0.742, n = 20, p<0.001)(Figure 3). A similar negative linear regression of dominance rank and pro-social tendency was found within the kin-pairs (t = −2.893, β = −0.715, n = 10, p = 0.02)(Figure 3). The effect of dominance rank may be due to either an individual's own rank position or to the rank distance with its partner. A multiple regression of pro-social tendency with both subject's rank and rank distance showed only a significant effect of the subject's rank position (t = −2.904, β = −0.565, n = 20, p = 0.01), yet no significant effect of rank distance (t = 1.472, β = 0.286, n = 20, p = 0.159). Therefore, we conclude that a subject's pro-social tendency depends on the absolute dominance rank of the subject itself.

## Discussion

This is, to our knowledge, the first study to show pro-sociality in a despotic monkey species. In line with the kin selection theory [23] and similar to capuchin monkeys [7], female long-tailed macaques behave pro-socially towards kin and are more pro-social towards kin than towards non-kin, reflecting the importance of their kin-relations [17], [but see 24]. Moreover, dominant individuals also provide benefits to non-kin others. These results suggest that pro-social behavior is not restricted to egalitarian species, and supports the hypothesis that all anthropoid primate species may share this behavior through common ancestry. Additional support, in the form of replication using other despotic species and comparisons with less despotic macaques, is needed. We emphasize, however, that further studies should be aware of relatedness and dominance rank as a possible interacting factor in any study of social cognitive capacities.

Machiavelli advised despotic leaders that ‘it is better to be feared than loved’. Our results, however, indicate that dominant animals actually provide benefits to others, while subordinates withhold them. Moreover, the absolute dominance rank of the subject, and not its rank position relative to that of its partner, determines its pro-social behavior, both among kin and in non-kin pairs. These results contrast with previous research on primates showing that subordinates give more grooming to dominants [e.g. 18], [19], presumably with tolerance or future support as a result. In addition, low-ranking individuals withheld their partner food. This may be part of a competitive strategy. Pro-social behaviour of only the dominants has been reported in humans too, where individuals in a dominant position behave more pro-socially than those in a subordinate position [25], [26]. Pro-sociality of a dominant individual may be a strategy to enhance or maintain status [20], [21]. Moreover, by being pro-social dominants may advertise their dominance, possibly convincing subordinates to accept the high-ranking individual's dominance and inhibiting rebellion of subordinates [27]. Therefore, this study suggests that dominant long-tailed macaques advertise their dominance position through pro-social behaviour, much like is expected in the handicap principle [28]. The handicap principle, however, specifically concerns behaviors that are costly for the actor, whereas in this experiment the actor has no costs. Whether long-tailed macaques would behave similarly when a cost to themselves is involved remains to be tested. Alternatively, it may be that not an individual's high dominance rank leads to its pro-social behaviour, but that the pro-social behaviour of an individual has lead it to achieve such a high dominance rank. For male long-tailed macaques it has already been suggested that not only their strength, but also their social capacities influence their position within a dominance hierarchy [29]. In contrast to males, females remain in the same group for the rest of their life [30], and have ‘family ranks’, since they inherit their rank from their mother [31]. However, family turnovers do occur [32], and pro-social behaviour of females may as well be a strategy to sustain their rank position. Nevertheless, our results indicate that dominant macaques are not just Machiavellian despots, but like benevolent leaders, also provide benefits to their subordinates.

## Materials and Methods

### Ethics Statement

The experiment has been conducted according to the directives of the Dutch experiments on animals act. The experiment was approved by the Ethics Committee of Utrecht University (DEC 2007.I.08.103) and thus complies with the Dutch law. To avoid any stress, the animals were never forced to participate. Consequently, the animals that were tested, participated voluntarily. The animals were, furthermore, never food or water deprived.

### Subjects and Test-setting

Ten male and ten female long-tailed macaques from a long-term, stable social group (colony of Utrecht University, The Netherlands) participated in this experiment. Experiments were carried out in familiar test chambers. The test-setting consisted of three connected chambers (110 cm×55 cm×80 cm) that were divided by two lexan transparent screens (Figure 1). The test apparatus was placed in front of the middle compartment. On the test apparatus were two handles that were connected to two separate sliding bars. Only one of these handles could be pulled per trial. At the beginning of each trial, four equally sized slices of banana simultaneously dropped on the two bars, one on each end of each bar. The monkey in the middle compartment then could pull either handle to move the bar with pieces of banana towards the compartments to grab and eat the treat from one side of the bar. The slice of banana at the other end of the bar was out of reach for the monkey in the middle compartment. However, if another monkey (kin or non-kin) was present in that outer compartment, it could take and eat the slice of banana. After the monkey(s) had taken their reward(s), the remaining banana slices were removed and a new trial was directly thereafter started. Pulling the handle that delivered the slice of banana to the other monkey too is considered as ‘pro-social’, while pulling the handle that delivers the banana to the front of an empty cage is termed ‘a-social’. It is important to note that the monkey in the middle compartment (the test-subject) always got a piece of banana, independent of which handle he/she pulled.

All subjects were already trained for a previous experiment [33] to pull in a bar baited with one reward for the pulling individual, and were equally efficient (100%) in this task. Moreover, for this previous experiment, the monkeys were also trained to be isolated in a test compartment alone or with a partner in a neighboring compartment that was separated from it by a lexan transparent screen. Therefore, the monkeys were familiar with a partner next to them, and had learned that this partner could not enter their compartment. Several days prior to testing all animals got access with several animals at a time to the apparatus that was baited at the two ends of each slide, such that all animals could experience that they could pull and obtain a reward, but that the second reward was out of reach and could be taken by another individual. The two days before testing, all subjects were trained without a partner in the final test-setting (i.e., in the middle test-compartment, between two empty compartments). During this final training they got 4 trials each day in which they had to choose between the two slides that were baited on each end and after they had made their choice the other slide was blocked.

### Conditions

Subjects were tested in an experimental and a control condition. Subjects were always in the middle chamber of the test setting. The subjects were first tested in the control condition, in which we determined the left/right preference of each subject without a partner. In the test condition all individuals were tested in the same way, but now with a same-sex partner sitting on the opposite side of their preferred side, as determined in the control condition. Both the control and the test condition consisted of twenty trials that, in order to retain the monkeys' motivation, were divided over two consecutive days, with ten trials on each day. The subjects did not differ significantly in the number of pro-social choices between the first ten trails and the second ten trials of the test condition (Wilcoxon signed ranks test: T^+^ = 110, n = 20, p_exact_ = 0.559), nor in their side preference between the first ten trials and the second ten trials of the control condition (Wilcoxon signed ranks test: T^+^ = 95, n = 20, p_exact_ = 0.707). Moreover, all animals completed all trials and were generally very motivated, since they almost always took the food (18–20 times). Consequently, there were no differences in motivation related to the dominance rank of the subjects. Furthermore, all animals generally ate the food items they retrieved or received from their partner. Aggressive behaviour was rare and, if present, directed at the experimenter. Finally, to avoid reciprocation, dyads were always novel. Ten (nine females and one male) out of the twenty subjects were counterbalanced tested with both a kin and a non-kin partner.

### Measures

To measure pro-sociality, the preference for the partner-side in the test condition was compared with the preference for the same side in the control condition. Moreover, to test the pro-sociality of each individual separately, we used chi-squared tests with the amount of left and right choices in the control condition as expected values and the amount of left and right choices in the test condition as the observed values. To compare pro-social tendencies between different individuals, we calculated pro-social tendencies by subtracting from the preference for the partner-side in the test condition the preference for the same side in the control condition. A positive pro-social tendency then shows pro-social behaviour, whereas a negative pro-social tendency shows a-social behaviour, since the tested individual actually withholds a reward from its partner.

### Analysis

The dominance hierarchy of the group was calculated using unidirectional submissive behaviour arranged in a socio-matrix. The dominance order most consistent with a linear hierarchy was determined with MatMan 1.1 (Landau's linearity index: h = 0.7204, p<0.001), indicating a significantly linear dominance hierarchy [34], [35]. Rank numbers were afterwards assigned with 1 for the most dominant individual and 35 for the most subordinate individual. Rank distance between a subject and its partner was calculated by subtracting the rank number of the subject from the rank number of its partner. For comparisons of preferences in the control- and test condition, and comparisons between pro-social tendencies, exact Wilcoxon signed ranks tests were used. Pro-social tendency was regressed on both dominance rank of the subject and rank distance between subject and partner. Residuals of each of these linear regression models do not differ significantly from a normal distribution. All reported P-values are two-tailed.
